# Retraction: RORα Suppresses Epithelial-to-Mesenchymal Transition and Invasion in Human Gastric Cancer Cells via the Wnt/β-Catenin Pathway

**DOI:** 10.3389/fonc.2020.607586

**Published:** 2020-10-28

**Authors:** Frontiers Editorial Office

Following publication, concerns were raised over the duplication of control figures throughout the manuscript. An investigation was conducted in accordance with Frontiers' guidelines and multiple flaws were found in the publication. The authors were not able to provide the raw data upon request. The authors respect the decision to retract the article.

